# Caterpillar-Induced Rice Volatile (E)-β-Farnesene Impairs the Development and Survival of *Chilo suppressalis* Larvae by Disrupting Insect Hormone Balance

**DOI:** 10.3389/fphys.2022.904482

**Published:** 2022-05-30

**Authors:** Lei Yang, Xiaomin Yao, Baosheng Liu, Yangchun Han, Rui Ji, Jiafei Ju, Xiaona Zhang, Shuwen Wu, Jichao Fang, Yang Sun

**Affiliations:** ^1^ College of Plant Protection, Nanjing Agricultural University, Nanjing, China; ^2^ Jiangsu Key Laboratory for Food and Safety-State Key Laboratory Cultivation Base of Ministry of Science and Technology, Jiangsu Academy of Agricultural Sciences, Institute of Plant Protection, Nanjing, China; ^3^ Laboratory for Conservation and Use of Important Biological Resources of Anhui Province, Anhui Provincial Key Laboratory of Molecular Enzymology and Mechanism of Major Diseases, College of Life Sciences, Anhui Normal University, Wuhu, China

**Keywords:** *Chilo suppressalis*, comparative transcriptome, (E)-β-farnesene, rice defense, terpene synthases

## Abstract

Significant research progress has recently been made on establishing the roles of *tps46* in rice defense. (E)-β-farnesene (Eβf) is a major product of *tps46* activity but its physiological functions and potential mechanisms against *Chilo suppressalis* have not yet been clarified. In the present study, *C. suppressalis* larvae were artificially fed a diet containing 0.8 g/kg Eβf and the physiological performance of the larvae was evaluated. In response to Eβf treatment, the average 2nd instar duration significantly increased from 4.78 d to 6.31 d while that of the 3rd instar significantly increased from 5.70 d to 8.00 d compared with the control. There were no significant differences between the control and Eβf-fed 4th and 5th instars in terms of their durations. The mortalities of the 2nd and 3rd Eβf-fed instars were 21.00-fold and 6.39-fold higher, respectively, than that of the control. A comparative transcriptome analysis revealed that multiple differentially expressed genes are involved in insect hormone biosynthesis. An insect hormone assay on the 3rd instars disclosed that Eβf disrupted the balance between the juvenile hormone and ecdysteroid levels. Eβf treatment increased the juvenile hormones titers but not those of the ecdysteroids. The qPCR results were consistent with those of the RNA-Seq. The foregoing findings suggested that Eβf impairs development and survival in *C. suppressalis* larvae by disrupting their hormone balance. Moreover, Eβf altered the pathways associated with carbohydrate and xenobiotic metabolism as well as those related to cofactors and vitamins in *C. suppressalis* larvae. The discoveries of this study may contribute to the development and implementation of an integrated control system for *C. suppressalis* infestations in rice.

## Introduction

In response to insect pest damage, plants typically synthesize and release large amounts of herbivore-induced plant volatiles (HIPVs) that favor interactions with the ambient environment. HIPVs attract predators and parasites that defend and protect plants against herbivores and prime defensive responses in neighboring plants (War et al., 2011; Dudareva et al., 2013; Turlings and Erb, 2018). HIPVs include green leaf volatiles (GLVs), terpenoids, nitrogenous compounds, and other aromatic compounds (Chen S. et al., 2020). GLVs include aldehydes, alcohols, and their esters and are released in large quantities after insects destroy fresh leaves (Ameye et al., 2018; Turlings and Erb, 2018). Terpenoids contain basic five-carbon isoprene units and are released by several plants in response to herbivory (Pichersky and Raguso, 2018). A few nitrogenous and aromatic compounds have also been implicated in the attraction of natural enemies (Turlings and Erb, 2018). Terpenoid emission rates and compositions widely vary among plant genotypes. Terpenoid quantities and ratios vary with arthropod attack and mediate tritrophic interactions among HIPVs (Ameye et al., 2018).

Terpene synthases (TPSs) regulate terpenoid biosynthesis (Bohlmann et al., 1998; Pazouki and Niinemets, 2016) and are generally upregulated in response to insect attacks (Capra et al., 2015; Mei et al., 2017). TPS upregulation usually enhances plant pest resistance (Hua et al., 2021; Liu et al., 2021). TPS overexpression promotes insect pest resistance in transgenic crops (Mei et al., 2017; Sun et al., 2017).

The rice stem borer *Chilo suppressalis* is a highly destructive insect pest in global rice production. It causes significant yield and economic losses and poses a severe threat to international food security (Chen G. et al., 2020; Ma et al., 2020). In rice, *tps46* is constitutively expressed even in the absence of herbivore attack (Sun et al., 2017). However, it was significantly upregulated in rice subjected to *C. suppressalis* infestation for 24 h (Sun et al., 2011; Sun et al., 2017). Thus, *tps46* might play a key role in the response of rice to *C. suppressalis* attack. *Tps46* silencing in rice plants increased susceptibility to *Rhopalosiphum padi* compared with Wt plants. By contrast, *tps46* overexpression enhanced relative *R. padi* resistance (Sun et al., 2017). Taken together, the foregoing findings suggest that *tps46*-mediated terpenoid synthesis is involved in the response of rice to *C. suppressalis*.

Eight volatile products are catalyzed by *tps46* in rice. Of these, limonene and Eβf were detected in all *tps46* interference, overexpression, and Wt rice lines. The other six volatiles were detected only in the *tps46* overexpression rice lines. The limonene and Eβf levels were significantly lower in the *tps46* interference lines and significantly higher in the *tps46* overexpression lines than the Wt lines (Sun et al., 2017). Therefore, limonene and Eβf may play central roles in the defense of rice against *C. suppressalis*. Nevertheless, the physiological functions and potential mechanisms of limonene and Eβf against *C. suppressalis* have not yet been elucidated.

Our pre-experiments indicated that Eβf had a stronger direct effect than limonene against *C. suppressalis* larva development and survival. Numerous studies have revealed that juvenile hormone (JH) and ecdysteroids are important regulators of insect growth and development (Liu et al., 2019). In addition, Eβf are sesquiterpenoids with structural similarities to juvenile hormone (Crock et al., 1997; Jindra et al., 2013; Gaddelapati et al., 2022). However, it is unclear whether Eβf impairs the development and survival of *C. suppressalis* larvae by affecting insect hormone. In this study, we focused on the direct effects and potential modes of action of Eβf against *C. suppressalis* larvae. The bioassays showed that Eβf strongly inhibited *C. suppressalis* development and was lethal in the early larval stages. A comparative transcriptome analysis disclosed multiple DEGs involved in insect hormone biosynthesis while GO database annotation indicated that they participated in “growth”, “developmental process”, and “reproduction”. The 3rd instar larval hormone assay showed that Eβf disrupted the balance between juvenile hormones and ecdysteroids. The Eβf treatment increased the titers of juvenile hormones but not ecdysteroids. Both classes of hormones are vital to insect development, metamorphosis, and reproduction (Dubrovsky, 2005; Santos et al., 2019; Song and Zhou, 2020). Thus, Eβf might influence development, metamorphosis, and reproduction in *C. suppressalis*. The results of the present study demonstrated that Eβf treatment impaired larval development in *C. suppressalis* primarily by disrupting insect hormone balance. The preceding discoveries may contribute to the development of integrated control methods and strategies for *C. suppressalis* infestations on rice.

## Materials and Methods

### Insect Rearing

*C. suppressalis* populations were collected from rice fields in Ruichang County, China, maintained at 25°C under LD photoperiod (14 h:10 h light:dark) and relative humidity of 80%, and fed an artificial diet. The artificial diet was made according to Han et al.’s method (Han et al., 2012).

### Eβf Treatment

Eβf was purchased from Sigma-Aldrich Corp. (St Louis, MO, United States). The optimal terpenoids and its concentration were determined according to prior research (Sun et al., 2017; Chen et al., 2021). The pre-experiment concentrations assayed were 0.08 g/kg, 0.8 g/kg, and 8 g/kg, and it was established that the artificial diet should contain a final concentration of 0.8 g/kg Eβf. The artificial diet was fed to the first instar larvae until death. Two replicates (24 larvae/replicate) were used in the pre-experiments while four replicates were used in the subsequent experiment.

### RNA Isolation and Library Preparation for Transcriptome Sequencing

*C. suppressalis* larvae were fed control diet or Eβf (0.8 g/kg) until the 3rd larval instar, when they were collected and preserved at −80°C until use. Total RNA was extracted from six 3rd instar *C. suppressalis* larvae with TRIzol reagent (Invitrogen, Carlsbad, CA, United States) according to the manufacturer’s instructions (three biological replicates per treatment). RNA degradation and contamination were monitored on 1% agarose gel. RNA purity was checked with an IMPLEN NanoPhotometer® (IMPLEN Inc., Westlake Village, CA, United States). RNA concentration was measured with a Qubit® RNA assay kit in a Qubit® 2.0 Fluorometer (Life Technologies, Waltham, MA, United States). RNA integrity was assessed with the RNA Nano 6000 assay kit and an Agilent Bioanalyzer 2100 system (Agilent Technologies, Santa Clara, CA, United States).

Three micrograms RNA was used as the input material to prepare each RNA sample. Sequencing libraries were generated with the NEB Next® Ultra™ RNA library prep kit for Illumina® (New England Biolabs, Ipswich, MA, United States/Illumina, San Diego, CA, United States) following the manufacturers’ recommendations. Index codes were added to attribute the sequences to each sample. Briefly, mRNA was purified from total RNA using poly-T oligo-attached magnetic beads. Fragmentation was conducted using divalent cations and at elevated temperature in NEB Next first-strand synthesis reaction buffer (5X; New England Biolabs). First-strand cDNA was synthesized with random hexamer primer and M-MuLV reverse transcriptase (RNase H-; Santa Cruz Biotechnology, Dallas, TX, United States). Second-strand cDNA was synthesized with DNA polymerase I and RNase H. The remaining overhangs were converted into blunt ends via exonucleases and polymerases. The 3’ ends of the DNA fragments were adenylated and a NEB Next adaptor (New England Biolabs) with hairpin loop structure was ligated to prepare for the hybridization. The library fragments were purified with an AMPure XP system (Beckman Coulter, Beverly, MA, United States) to select 150–200-bp cDNA fragments. Three microliters USER enzyme (New England Biolabs) was combined with size-selected, adaptor-ligated cDNA at 37°C for 15 min followed by 95°C for 5 min before PCR. The latter was performed with Phusion high-fidelity DNA polymerase (Thermo Fisher Scientific, Waltham, MA, United States), universal PCR primers, and Index (X) primer. The PCR products were purified in an AMPure XP system and library quality was assessed in an Agilent Bioanalyzer 2100 system (Agilent Technologies).

The index-coded samples were clustered in a cBot cluster generation system using TruSeq PE cluster kit v. 3-cBot-HS (Illumina) according to the manufacturer’s instructions. The library preparations were then sequenced on an Illumina HiSeq platform (Illumina) and 150-bp paired-end reads were generated.

Raw data (raw reads) in fastq format were processed through in-house perl scripts. Clean data (reads) were obtained by removing the reads containing adapters and poly-N and the low-quality reads from the raw data. Q20, Q30, GC content, and sequence duplication level were calculated for the clean data. All subsequent analyses were based on high-quality clean data.

Reference genome and gene model annotation files were directly downloaded from the genome website. The reference genome index was built and paired-end clean reads were aligned to the reference genome with HISAT2 (http://daehwankimlab.github.io/hisat2/) (Kim et al., 2019). HISAT2 was selected as the mapping tool as it can generate a database of splice junctions according to the gene model annotation file. Thus, it yields better mapping results than other non-splice mapping tools. StringTie (https://github.com/gpertea/stringtie) was used to construct and identify both known and heretofore unidentified transcripts in the HISAT2 alignment results (Pertea et al., 2015).

### Quantification of Gene Expression Levels

StringTie was used to enumerate the reads mapped to each gene. Clean data were mapped back onto the reference genome. Read counts for each gene were obtained from the mapping results. FPKM (expected number of fragments per kilobase of transcript sequence per million base pairs sequenced) for each gene were calculated based on the length of the gene and the read counts mapped to it. FPKM simultaneously considers the effects of sequencing depth and gene length on the reads count and is the most commonly used method for estimating gene expression levels at present (Trapnell et al., 2010).

### Differential Expression Analysis

A differential expression analysis of both conditions/groups was performed using the DESeq package (v. 1.10.1) in R (R Core Team, Vienna, Austria) (Anders and Huber, 2010). DESeq runs statistical routines to generate differential digital gene expression data using a model based on the negative binomial distribution. The resulting *p* values were subjected to Benjamini and Hochberg correction to control the false discovery rate (FDR). Genes with adjusted *p*-value < 0.05 were designated as differentially expressed.

### Gene Functional Annotation

Gene function was annotated based on Nr (NCBI non-redundant protein sequences), Nt (NCBI non-redundant nucleotide sequences), Pfam (Protein family); KOG/COG (Clusters of Orthologous Groups of proteins), Swiss-Prot (a manually annotated and reviewed protein sequence database), KO (KEGG Ortholog database), and GO (Gene Ontology).

### GO Enrichment Analysis of Differentially Expressed Genes

Gene Ontology (GO) enrichment analysis of the DEGs was performed in the GOseq package of R and was based on the Wallenius non-central hypergeometric distribution (Young et al., 2010) which corrects for gene length bias.

### KEGG Pathway Enrichment Analysis of Differentially Expressed Genes

KOBAS 3.0 software (http://bioinfo.org/kobas/genelist/) was used to determine the statistical enrichment of the DEGs in the KEGG pathways (Bu et al., 2021).

### Quantitative PCR

Twelve candidate genes were randomly selected for quantification to verify the accuracy of the transcriptome data. The same RNA samples used for transcriptome sequencing were also used for qPCR. The RNA samples were reversed-transcribed into cDNA as templates (Takara). Each sample included three biological replicates. The qPCR reactionswere performed using a TB Green™Premix Ex Taq™kit (TaKaRa) and run on a LightCycler® 480 Instrument II Real-Time PCR System (Roche, Basel, Switzerland). Melting curves were plotted to examine amplification specificity. Standard curves were plotted using alinear regression model, and PCR efficiency was calculated according to the slope of the standard curve. The qPCR-specific primers for the twelve candidate genes were designed with Primer Premier v. 6.0 (PREMIER Biosoft, Palo Alto, CA, United States). EF1 was amplified as the standard control (Xu et al., 2017). Relative expression was quantitated by the 2^−ΔΔCt^ method (Livak and Schmittgen, 2001). The PCR primers used in this study are shown in Supplementary Table S3.

### Juvenile Hormone and Ecdysteroid Assays

Whole insects were homogenized in phosphate buffer and centrifuged at 3,000 rpm at 4°C for 10 min. The supernatants were used for juvenile hormone and ecdysteroid quantification with separate insect juvenile hormone and ecdysone ELISA test kits (Renjie Biotechnology Co. Ltd., Shanghai, China) according to the manufacturer’s instructions.

### Data Analysis

The Mann-Whitney U test was used to identify the variations in average instar duration and the levels of juvenile hormones and ecdysteroids in *C. suppressalis* larvae. One-way analysis of variance (ANOVA) was used to determine the differences between treatments in terms of their mortalities. Statistical analyses were run in SPSS v. 24.0 (IBM Corp., Armonk, NY, United States).

## Results

### Eβf Impairs *C. suppressalis* Larval Development and Survival

**FIGURE 1:**
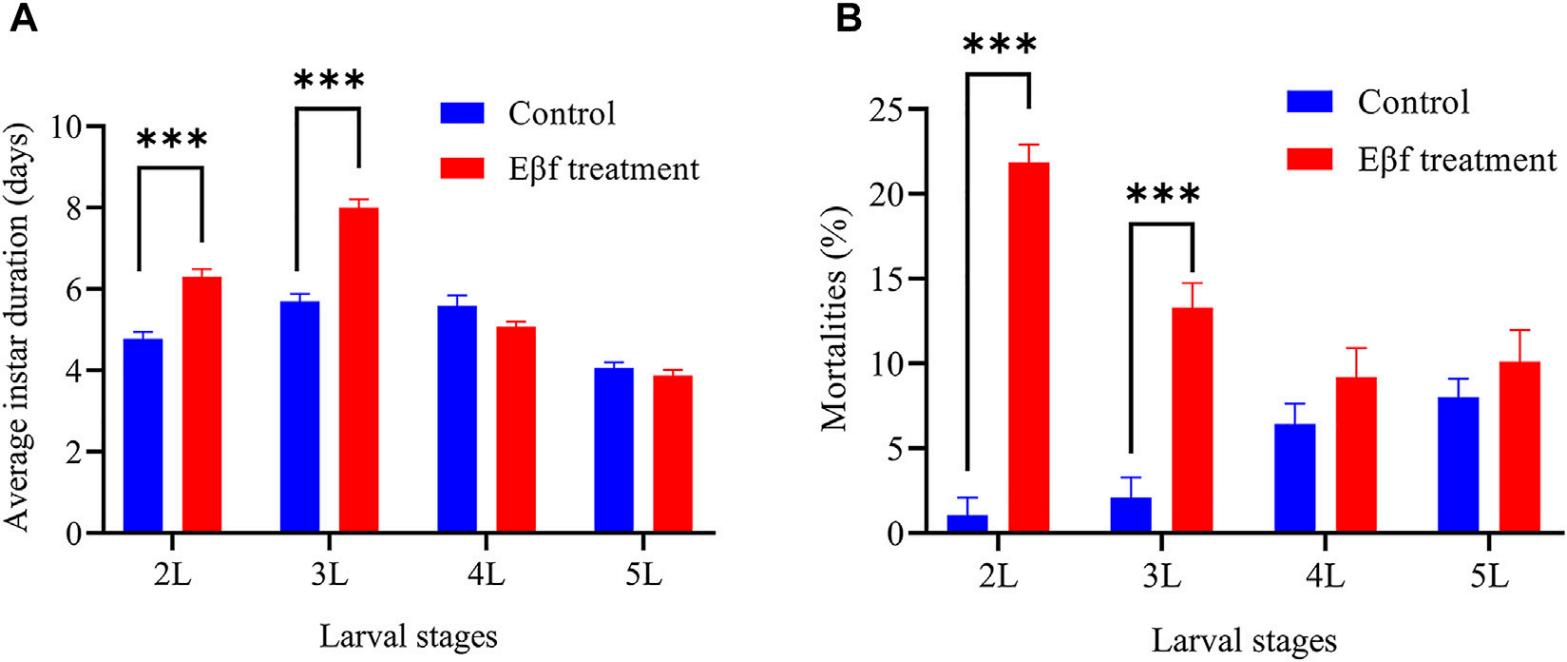
Average instar durations and mortalities in *C. suppressalis* larvae fed artificial diet containing Eβf. **(A)** Average instar duration. **(B)** Mortalities. Values are means ± SEM of four replicates (*n* = 24; N = 96). Asterisks indicate significant differences: *p < 0.05; **p < 0.01; ***p < 0.001 (Mann-Whitney *U* test).

Pre-experiments revealed that Eβf had a stronger direct effect than limonene on *C. suppressalis* larval development and survival (Supplementary Figures 1–6). Figure 1A shows that the average duration of the 2nd instar larvae significantly increased from 4.78 d to 6.31 d after the Eβf treatment (*p* < 0.001). The Eβf treatment also significantly prolonged the 3rd instar larvae from 5.70 d to 8.00 d (*p* < 0.001). There were no significant differences between the Eβf treatment and control in terms of the durations of the 4th and 5th instar larvae (*p* > 0.05). The foregoing data indicated that Eβf strongly inhibited the development of *C. suppressalis* at its earlier larval stages. The mortalities of the 2nd and 3rd instar larvae were 21.00-fold and 6.39-fold higher, respectively, than those of the control (*p* < 0.001). However, there were no significant differences between the Eβf treatment and the control in terms of the mortalities of the 4th and 5th instars (*p* > 0.05) (Figure 1B). The preceding data indicated that Eβf was lethal to *C. suppressalis* at its earlier larval stages. We also found that many of the *C. suppressalis* still surviving at the final larval stages permanently failed to pupate after Eβf treatment. Hence, Eβf impairs development and survival in *C. suppressalis* larvae and Eβf might efficaciously protect rice against *C. suppressalis*.

### Transcriptome Sequencing and Assembly

The transcriptomes of the 3rd instar larvae subjected to Eβf and solvent were sequenced on the Illumina HiSeq 2000 platform. From six *C. suppressalis* larvae, we obtained 52.71 Gb clean data with Q30 >91.55% after filtering the raw reads. The GC content was in the range of 48.66–54.42% and all read alignments were >89.00% (Supplementary Table S1). The clean reads were aligned with HISAT2 to the reference genome (https://www.ncbi.nlm.nih.gov/genome/?term=Chilo+suppressalis). We used StringTie to construct and identify known and novel transcripts from the HISAT2 alignment results. The clean data were assembled into 38,698 genes (Supplementary Table S1).

### Functional Annotation

The genes were annotated by a BLAST search against the COG, GO, KEGG, KOG, Pfam, SwissProt, and Nr databases. The cut-off E-value was 1E-5. Of all genes, 24,036 (62.11%) were annotated to the foregoing databases (Supplementary Table S2). Of these, the major genes (99.52%) were annotated against the Nr database and >50% of them were long (≥1,000 bp) (Supplementary Table S2). A total of 14,591 genes had matches in the Pfam database followed by the SwissProt and KOG databases. The annotated genes had few matches in the KEGG (26.36%), COG (27.25%), or GO (30.97%) databases (Supplementary Table S2).

### Differential Expression Analysis

**FIGURE 2:**
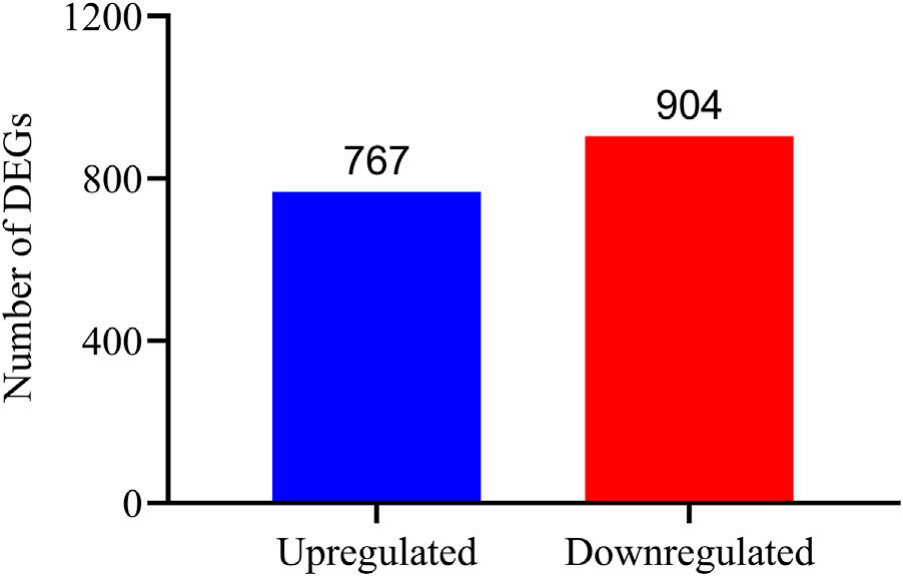
Gene expression profiles of control and Eβf treatment on 3rd instar *C. suppressalis* larvae. Blue bar represents 767 significantly upregulated genes while red bar represents 904 significantly downregulated genes in Eβf treatment compared with expression levels of same in control.

Analysis of DEGs between the Eβf treatment and the control was performed using the DESeq v. 1.10.1 package in R. Genes with adjusted *p* < 0.05 were designated as DEGs. In total, 1,671 and 270 DEGs were identified in the 3rd instar *C. suppressalis* Eβf and control larvae, respectively. There were 767 upregulated and 904 downregulated genes (Figure 2). Twelve candidate genes were randomly selected and quantitated to validate the DEGs identified by transcriptome sequencing. The trends in the expression of these DEGs were consistent with the results of the transcriptome sequencing. Thus, the output of the latter was credible (Figure 6).

### GO Classification

**FIGURE 3:**
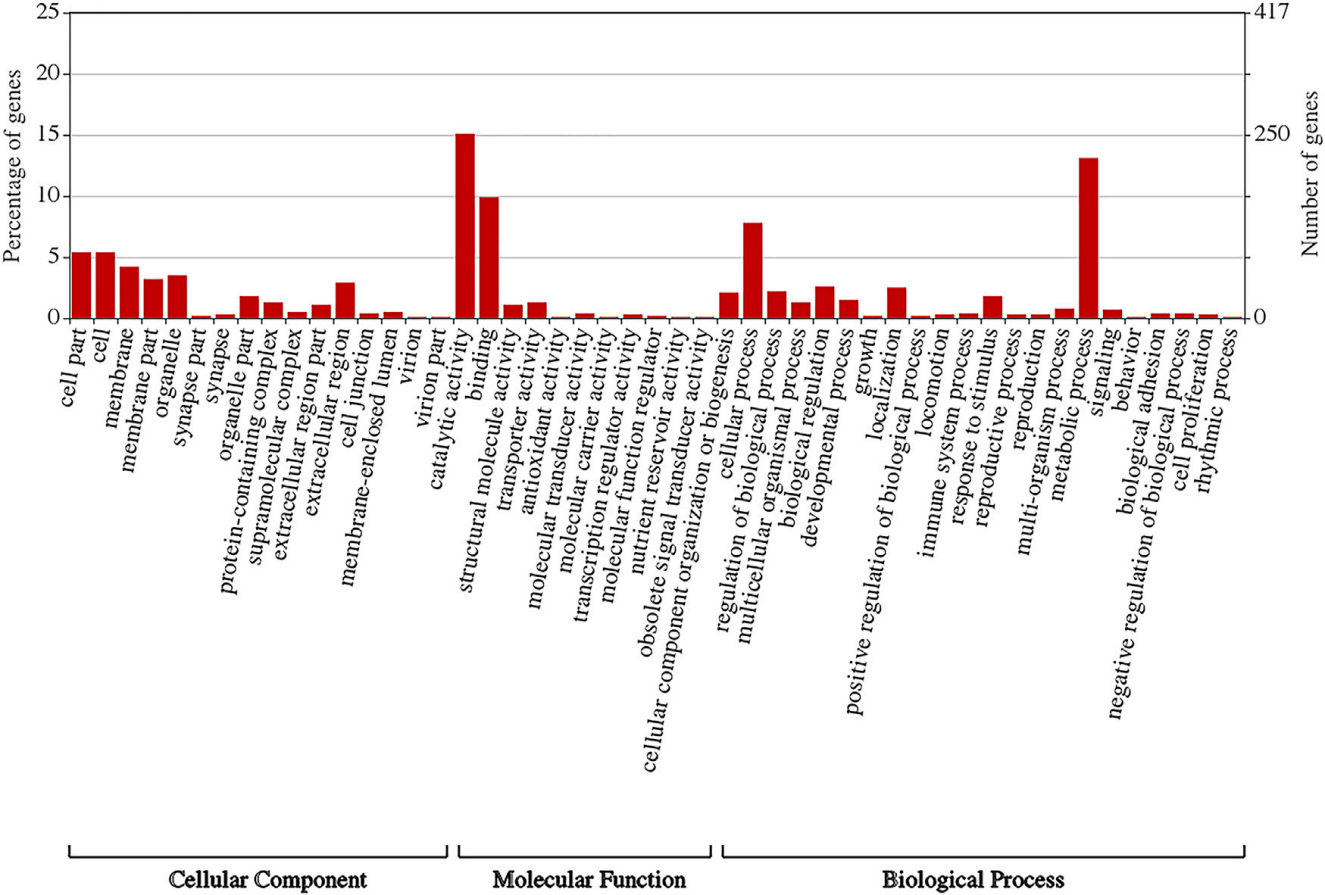
Gene Ontology (GO) classification of differentially expressed genes (DEGs). Gene Ontology (GO) enrichment analysis of DEGs. Top three enriched terms in cellular component (CC) category were cell”, “cell part”, and “membrane”. Terms “catalytic activity”, “binding”, and “transporter activity” were dominant in molecular function (MF) category. In biological process (BP) category, major GO terms were “metabolic process” and “cellular process”.

Gene Ontology (GO) is a universally standardized gene functional classification system that annotates numerous genes and their products and analyzes their functions. The GO consortium provides a standardized hierarchical vocabulary (GO terms) to describe gene product functions. GO also classifies genes into functional categories under general sections of molecular function, biological process, and cellular component. To clarify the main functions of all the DEGs, 418 of them were assigned to 46 GO terms (Figure 3).

Within the cellular component category, the major GO terms were “cell”, “cell part”, “membrane”, “membrane part”, “extracellular region”, “organelle part”, “protein-containing complex”, and “extracellular region part”. The terms “supramolecular complex”, “membrane-enclosed lumen”, “cell junction”, “synapse”, “synapse part”, “virion”, and “virion part” had low values (Figure 3).

Within the molecular function category, “catalytic activity” had a high value followed by “binding”, “transporter activity”, “structural molecule activity”, “molecular transducer activity”, “transcription regulator activity”, “molecular function regulator”, “antioxidant activity”, “nutrient reservoir activity”, and “molecular carrier activity” (Figure 3).

Within the biological process category, the major GO terms were “metabolic process” and “cellular process” followed by “biological regulation”, “localization”, “cellular component organization or biogenesis”, “response to stimulus”, “multicellular organismal process”, “developmental process”, “multi-organism process”, “signaling”, “biological adhesion”, “reproduction”, “reproductive process”, “locomotion”, “immune system process”, “behavior”, “growth”, “rhythmic process”, “detoxification”, and “cell proliferation” (Figure 3).

### KEGG Analysis

**FIGURE 4:**
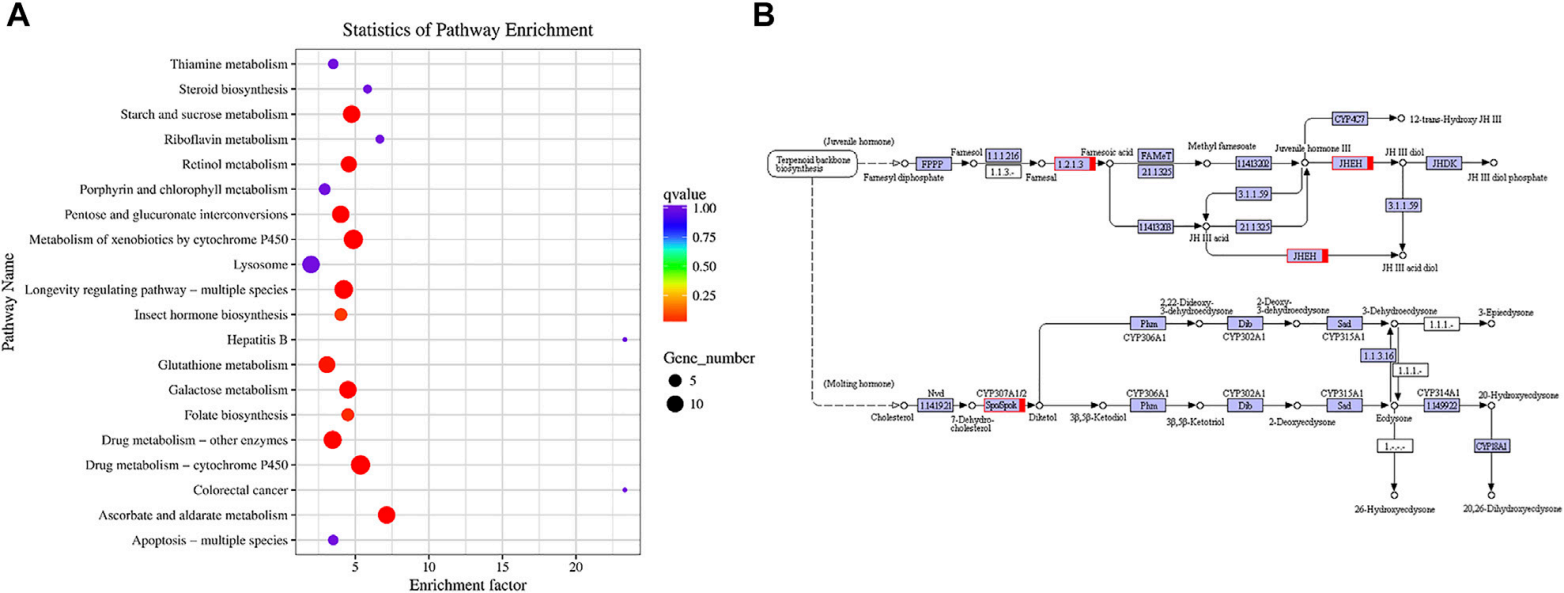
DEG pathway enrichment analysis. **(A)** Top 20 pathway enrichment analysis of differentially expressed genes. **(B)** Insect hormone biosynthesis pathway. Pathways were identified based on number of enriched genes, *p*-value, and rich factor.

KEGG (Kyoto Encyclopedia of Genes and Genomes) is a knowledge base applied in the systematic analysis of gene functions. It links genomic and higher-order functional information. The latter is stored in the PATHWAY database and supplemented by a set of ortholog group tables of information about conserved subpathways (pathway motifs). The latter are often encoded by positionally coupled genes on the chromosome and are especially useful in predicting gene functions. Thus, we performed a KEGG pathway analysis of the DEGs. The top 20 enriched pathways are shown in Figure 4A.

The top enriched pathways were “Ascorbate and aldarate metabolism” followed by “Drug metabolism-cytochrome P450”, “Xenobiotic metabolism-cytochrome P450”, “Longevity regulating pathway-multiple species”, “Starch and sucrose metabolism”, “Galactose metabolism”, “Pentose and glucuronate interconversions”, “Retinol metabolism”, “Drug metabolism-other enzymes”, “Glutathione metabolism”, “Folate biosynthesis”, and “Insect hormone biosynthesis” (Figure 4A). All of these belong to the Metabolism category except for “Longevity regulating pathway-multiple species” which belongs to the Organismal Systems category.

Figure 4B shows that multiple genes regulate juvenile hormone and ecdysteroid biosynthesis and degradation. Here, all these genes were upregulated. Enzyme “1.2.1.3” is aldehyde dehydrogenase which is the main step in converting farnesal to farnesol. Juvenile hormone epoxide hydrolase (JHEH) degrades JH III by hydrating its epoxide moiety to JH III diol. CYP307A1 participates in ecdysone synthesis. Abnormal expression of the preceding genes may cause hormonal imbalance in *C. suppressalis* larvae.

### Eβf Disrupting Insect Hormone Balance

**FIGURE 5:**
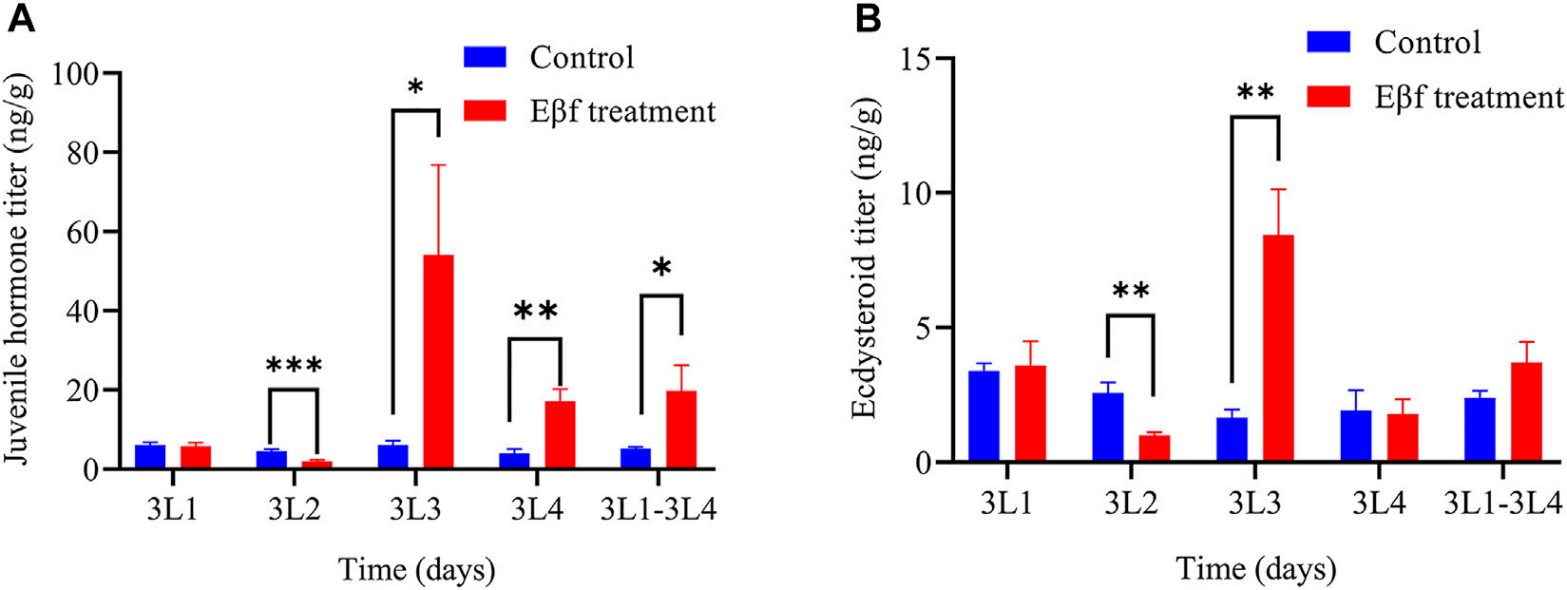
Effect of Eβf treatment on insect hormone titers in 3rd instar *C. suppressalis* larvae. **(A)** Juvenile hormone titer. **(B)** Ecdysteroid titer. Values are means ± SEM of three replicates. Asterisks indicate significant differences: *p < 0.05; **p < 0.01; ***p < 0.001 (Mann-Whitney *U* test).

Multiple DEGs were involved in the insect hormone biosynthesis pathway. Hence, we evaluated the juvenile hormone and ecdysteroid levels. The effects of Eβf treatment on the juvenile hormone and ecdysteroid levels in *C. suppressalis* larvae are shown in Figure 5. The Eβf treatment significantly increased the juvenile hormone levels in 3-day-old and 4-day-old 3rd instar larvae, significantly reduced it in 2-day-old 3rd instar larvae (Figure 5A). For each sample, ecdysteroid levels were also measured at the same time. The Eβf treatment significantly increased ecdysteroid levels in 3-day-old 3rd instar larvae, significantly reduced it in 2-day-old 3rd instar larvae (Figure 5B). Additionally, the Eβf treatment the entire time period also resulted in higher the juvenile hormone levels compared to control the entire time period (Figure 5A). Conversely, ecdysteroid levels were not (Figure 5B). To sum up, the Eβf treatment significantly increased the juvenile hormone but not the ecdysteroid levels at the 3rd larval stage.

## Discussion

The acyclic sesquiterpene Eβf is regulated by *tps46* (Sun et al., 2017). Previous studies on Eβf function focused on its roles in indirect defense (Verheggen et al., 2012; Li et al., 2019). Eβf is the main component of aphid alarm pheromones. It interrupts aphid feeding and causes conspecifics in the vicinity to become agitated and/or disperse from the host plant (Sun et al., 2017; Sun and Li, 2017). Ladybird beetles are attracted to young stage-2 pyrethrum flowers emitting the highest levels of the purest Eβf (Li et al., 2019). However, the foregoing responses are all indirect Eβf defenses. Here, we report the direct inhibitory effects of Eβf on 2nd and 3rd instar larval development and mortality in *C. suppressalis* (Figure 1).

**FIGURE 6:**
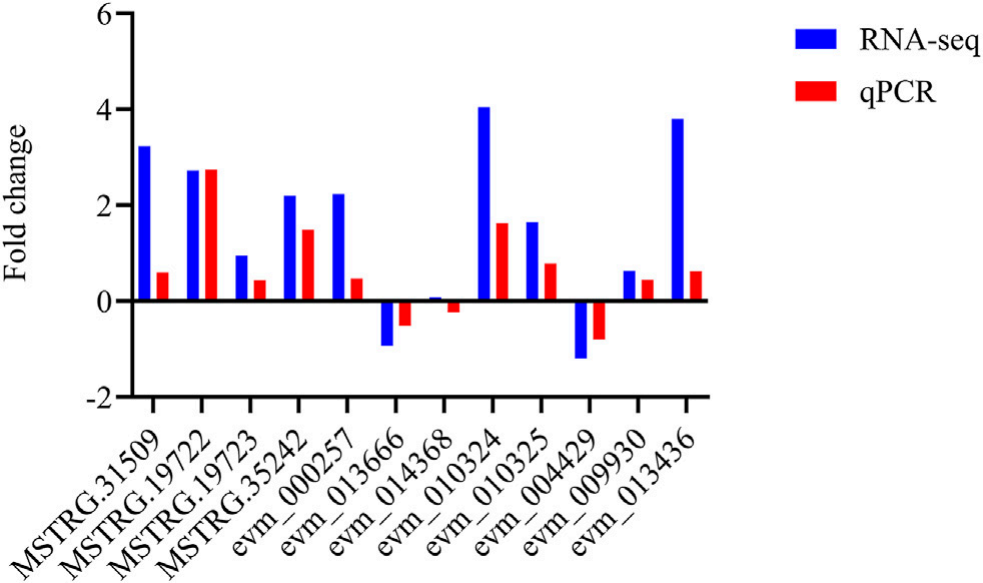
Expression of twelve randomly selected genes validated by qPCR. Validation of RNA-Seq data by qPCR. Twelve candidate genes were randomly selected for quantification. Column height is proportional to fold change of gene expression. Fold change is positive for upregulated genes and negative for downregulated genes. Values are means.

A comparative transcriptome analysis showed that multiple DEGs regulate insect hormone biosynthesis. A GO database annotation indicated that these DEGs participate in “growth”, “developmental process”, and “reproduction”. The hormone assay on the 3rd larval *C. suppressalis* instars showed that Eβf caused an imbalance between juvenile hormones and ecdysteroids. Eβf treatment increased juvenile hormone but not ecdysteroid titers. Juvenile hormones and ecdysteroids play pivotal roles in insect development, metamorphosis, and reproduction (Dubrovsky, 2005; Santos et al., 2019; Song and Zhou, 2020). A proper balance between juvenile hormone and ecdysteroids is of a paramount importance for insect development (Gruntenko and Rauschenbach, 2008; Rauschenbach et al., 2008; Meiselman et al., 2017; Liu et al., 2018). In *Drosophila*, it has been reported that there is a mechanism of reciprocal regulation of juvenile hormone and ecdysteroids which is responsible for their proper balance. An experimental rise in juvenile hormone titre leads to a rise in ecdysteroids in *Drosophila virilis* (Rauschenbach et al., 2007), and an experimental rise in ecdysteroids titre results in a dose-dependent decrease of juvenile hormone degradation (Gruntenko et al., 2003; Gruntenko et al., 2005). Accordingly, a steep decrease of ecdysteroids titre as a result of a mutation leads to an increase in juvenile hormone degradation in *Drosophila melanogaster* (Rauschenbach et al., 2008). Insect hormone imbalance leads to dramatic changes in insect development, metamorphosis, oogenesis and fecundity (Gruntenko and Rauschenbach, 2008; Rauschenbach et al., 2008; Ogihara et al., 2015). Insect hormone imbalance caused by RNAi-based knockdown of *sad*, which involved in the last step of ecdysteroids biosynthesis caused a longer developmental duration and lower pupation of 4th instar larvae, as well as caused shorter ovarioles and fewer fully developed eggs (Peng et al., 2019). Similar results were observed after RNAi-based knockdown of JHE (juvenile hormone esterase) on *Sesamia nonagrioides* (Kontogiannatos et al., 2013). Hence, Eβf might influence *C. suppressalis* development, metamorphosis, and reproduction. Our data disclosed that Eβf treatment impaired *C. suppressalis* larval development and survival. Previous research has revealed that Eβf mimics the action of juvenile hormone III in certain insect species (Crock et al., 1997). In our study, Eβf treatment increased the juvenile hormones titers but not those of the ecdysteroids. This result fits nicely with previous research. Therefore, the most reliable explanation is that Eβf also mimics the action of juvenile hormone in *C. suppressalis*. In addition, Eβf is also toxic to aphid and whiteflies (Crock et al., 1997). In this aspect, our results are consistent with these previous findings. The application of juvenile hormone analog insecticides to larvae also impaired their development and survival (Hu et al., 2019; Hu et al., 2020). We did not systematically investigate *C. suppressalis* metamorphosis and reproduction here as it was difficult to sample sufficient pupae and maintain the population for these purposes. Our findings demonstrated that Eβf strongly affected *C. suppressalis*. We discovered that Eβf impairs larval development and mortality in *C. suppressalis* by disrupting insect hormone balance. The qPCR results disclosed DEG trends that were consistent with those obtained by RNA-Seq (Figure 6). Both assays established that multiple DEGs in the insect hormone biosynthesis pathway were upregulated.

The KEGG database showed that the response of *C. suppressalis* to Eβf was associated with carbohydrates (ascorbate and aldarate, starch and sucrose, and galactose metabolism as well as pentose-glucuronate interconversions), xenobiotics (drug metabolism-cytochrome P450; xenobiotic metabolism-cytochrome P450; and drug metabolism-other enzymes), and cofactors and vitamins (retinol metabolism and folate biosynthesis) (Figure 4A). Previous studies reported that ascorbate is essential for several different metabolic reactions in animals. It is an essential water-soluble vitamin and cofactor in humans and a vital antioxidant that scavenges reactive oxygen species (ROS) and improves disease resistance (Ballaz and Rebec, 2019; Liu et al., 2020). Here, all 11 upregulated genes were identified among the DEGs. The Eβf treatment may enhance ascorbate metabolism in *C. suppressalis*. Eβf toxicity might be associated with the stimulation of ROS production, lipid peroxidation, and perturbation of the total antioxidant capacity. Therefore, the reduction of oxidative stress mediated by the upregulation of ascorbate metabolism could be an important molecular mechanism by which *C. suppressalis* responds to Eβf. We also observed that multiple upregulated and downregulated genes were involved in starch and sucrose metabolism, galactose metabolism, and pentose-glucuronate interconversion.

Xenobiotic metabolism is essential in insects as it enables them to neutralize and eliminate toxins that could interfere with vital biochemical processes (Schuler, 2011; Chen G. et al., 2020; Palli, 2020). Here, multiple P450 genes involved in cytochrome P450-mediated drug and xenobiotic metabolism were upregulated. P450s are associated with reciprocal adaptation of insect detoxification genes in response to plant biosynthetic genes. This response is driven by the co-evolution of herbivores and their chemically defended plant hosts (Schuler, 2011; Calla et al., 2017). These P450s may participate in Eβf biodegradation and metabolism. Furthermore, xanthine dehydrogenase/oxidase, glucuronosyltransferase, and *β*-glucuronidase were upregulated and identified in other enzymatic drug metabolism pathways.

We also detected alterations in the pathways regulating retinol metabolism and folate biosynthesis. Retinol influences cell differentiation, proliferation, and apoptosis and plays important roles in a wide range of physiological processes (Doldo et al., 2015). Retinoid biosynthesis maintains a condition that is conducive to regenerative growth in *Drosophila* (Halme et al., 2010). Tetrahydrofolate and its derivatives (folates) are essential in nearly all living organisms (Gorelova et al., 2019). Fruit fly larvae with low folate intake had significantly lower growth and DNA synthesis rates than normal controls (Blatch et al., 2015). The observed delay in the growth and development of the 3rd instar larvae fed Eβf might be related to low folate intake.

It is uncertain whether the biological activity of Eβf is consistent with that of juvenile hormones. Farnesol is a sesquiterpenoid precursor of insect juvenile hormones but its activity was far weaker than that of partially purified cecropia moth (*Hyalophora cecropia*) extracts. Hence, farnesol is probably not true insect juvenile hormone (Yamamoto and Jacobson, 1962; Staal, 1975; He et al., 2011). Rather, it might be a precursor or analog of juvenile hormone that simply increases juvenile hormones titers, thereby creating an imbalance between the juvenile hormone and ecdysteroid levels. This dysregulation impairs larval development and increases mortality in *C. suppressalis*.

## Conclusion

The bioassays and transcriptome data suggested that Eβf impairs development and increases mortality in *C. suppressalis* larvae by disrupting insect hormone balance. The qPCR showed that Eβf induces numerous DEGs regulating insect hormone biosynthesis. The foregoing findings will help elucidate the ecological roles of Eβf plays in the direct defense of rice against insect pest herbivory. Eβf altered the pathways associated with carbohydrate and xenobiotic metabolism as well as cofactors and vitamins in *C. suppressalis* larvae. Thus, transcriptomics is invaluable in clarifying the potential mechanisms by which Eβf delays development and increases mortality in *C. suppressalis* larvae. The discoveries of the present study will facilitate the development of integrated control managements against *C. suppressalis* infestations in rice.

## Data Availability

The data presented in the study are deposited in the Gene Expression Omnibus (GEO) database, accession number GSE179532.
