# Supplementary material for: Caterpillar-Induced Rice Volatile (E)-β-Farnesene Impairs the Development and Survival of Chilo suppressalis Larvae by Disrupting Insect Hormone Balance
Source: Front Physiol. 2022 May 30;13:904482. doi: 10.3389/fphys.2022.904482 (PMC9196309; doi:10.3389/fphys.2022.904482)
Supplement: Supplementary file 1 [file DataSheet1.PDF]

Supplementary Material

1 Supplementary Figures and Tables

1.1 Supplementary Figures

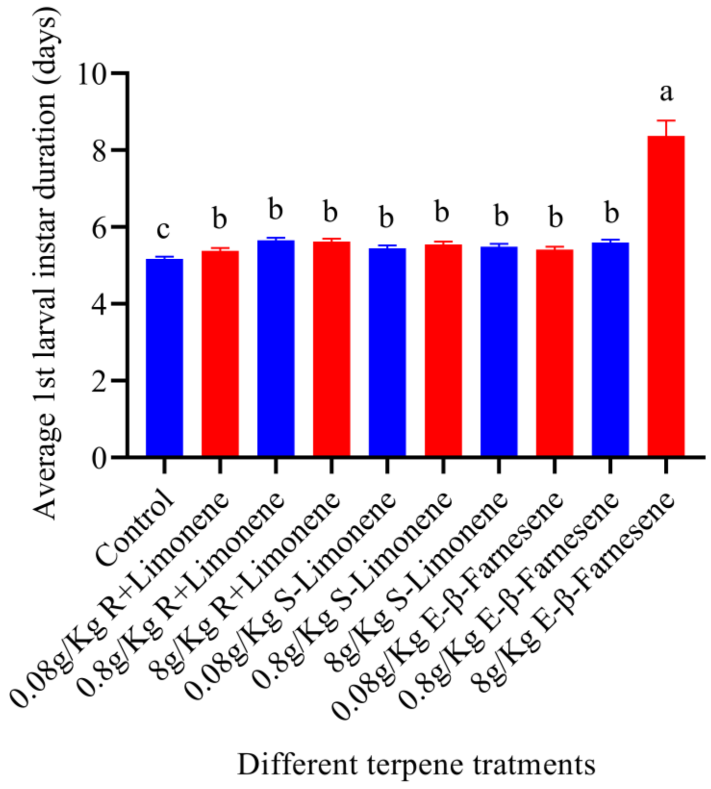

Supplementary Figure 1. Average instar duration in 1st instar *C. suppressalis* larvae fed artificial diet containing different terpene. Values are means ± SEM of two replicates (n = 24, N = 48). Different lowercase letters indicate statistically significant ($P < 0.05$) difference (The Kruskal Wallis test).

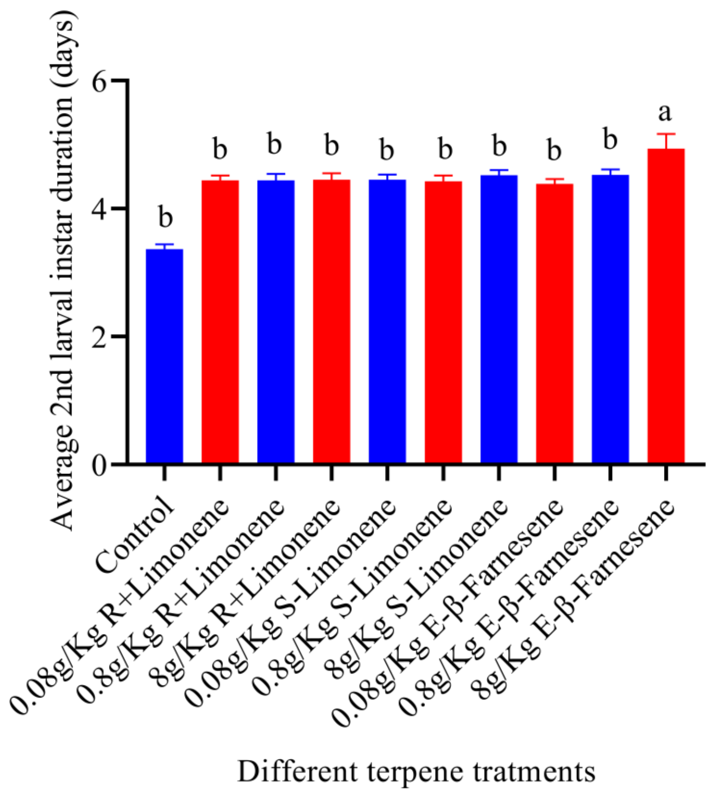

Supplementary Figure 2. Average instar duration in 2nd instar *C. suppressalis* larvae fed artificial diet containing different terpene. Values are means ± SEM of two replicates (n = 24, N = 48). Different lowercase letters indicate statistically significant (P < 0.05) difference (The Kruskal Wallis test).

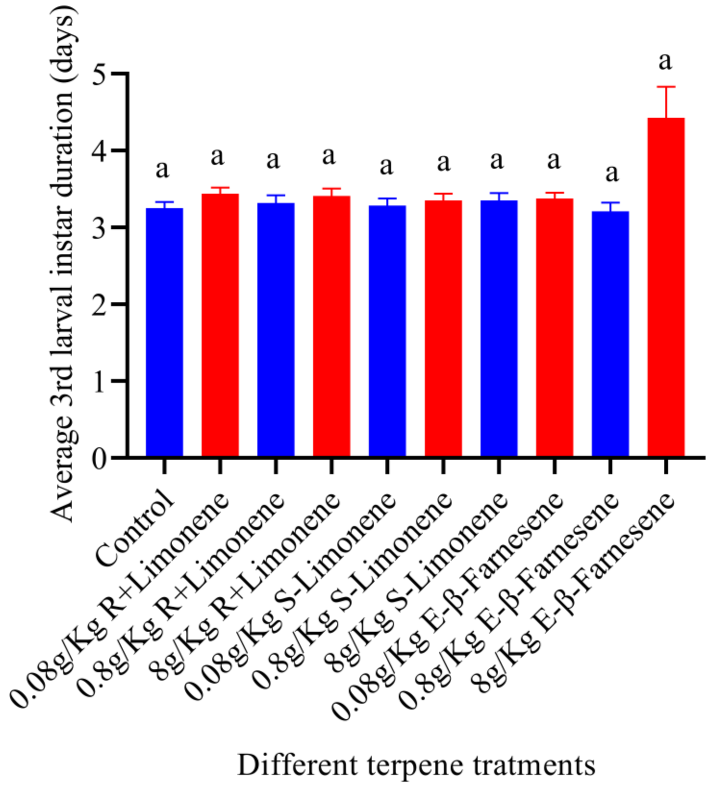

Supplementary Figure 3. Average instar duration in 3rd instar *C. suppressalis* larvae fed artificial diet containing different terpene. Values are means ± SEM of two replicates (n = 24, N = 48). Different lowercase letters indicate statistically significant ($P < 0.05$) difference (The Kruskal Wallis test).

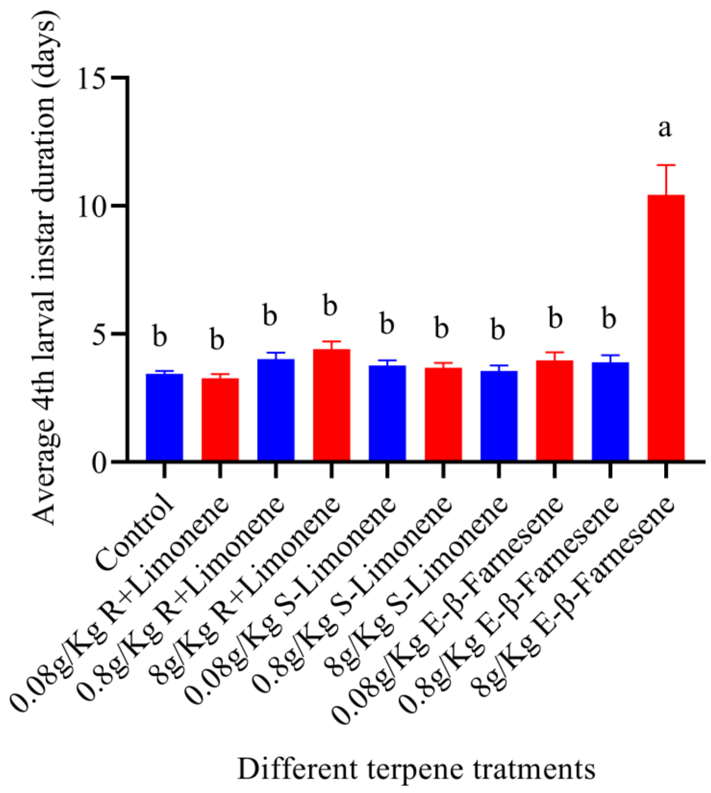

Supplementary Figure 4. Average instar duration in 4th instar *C. suppressalis* larvae fed artificial diet containing different terpene. Values are means ± SEM of two replicates (n = 24, N = 48). Different lowercase letters indicate statistically significant (P < 0.05) difference (The Kruskal Wallis test).

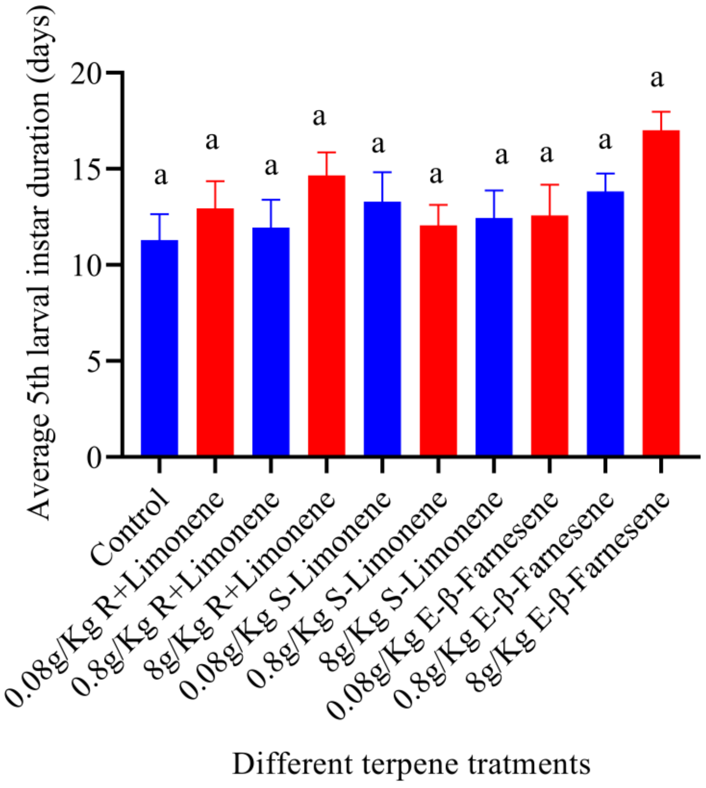

Supplementary Figure 5. Average instar duration in 5th instar *C. suppressalis* larvae fed artificial diet containing different terpene. Values are means ± SEM of two replicates (n = 24, N = 48). Different lowercase letters indicate statistically significant ($P < 0.05$) difference (The Kruskal Wallis test).

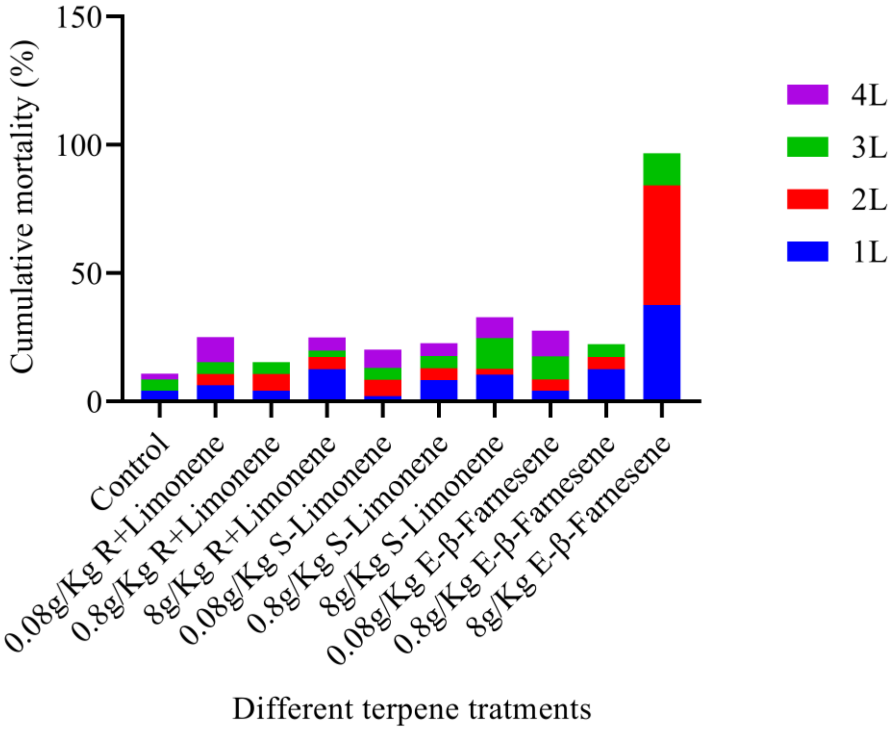

Supplementary Figure 6. The effect of limonene and Eβf on *C. suppressalis* larvae mortality rates. The blue bar represent mortality rates of *C. suppressalis* 1st instar larvae, the red bar for 2nd instar larvae, the green for 3rd instar larvae and the purple for 4th instar larvae.

1.2 Supplementary Figures

Supplementary Table 1. Transcriptome sequencing data and assembly

Sample	Clean data	No. bases	GC (%)	Q30 (%)	No. aligned reads
TYC1	26,970,894	8.09E+09	50.48	94.55	49,854,726 (92.42%)
TYC2	33,205,800	9.96E+09	50.69	94.58	61,096,065 (92.00%)
TYC3	27,219,428	8.17E+09	54.42	91.98	48,453,034 (89.00%)
TFN1	40,041,578	1.2E+10	51.79	94.88	75,239,649 (93.95%)
TFN2	34,212,787	1.03E+10	51.42	94.84	63,564,292 (92.90%)
TFN3	27,008,118	8.1E+09	48.66	92.59	48,201,918 (89.24%)
Total No. genes = 38,698					

Supplementary Table 2. Functional annotation of the *C. suppressalis* transcriptome

Anno_Database	Annotated_Number	300 ≤ length < 1,000	Length ≥ 1,000
COG_Annotation	6,549	1,993	4,385
GO_Annotation	7,445	2,889	4,173
KEGG_Annotation	6,335	2,227	3,860
KOG_Annotation	13,017	4,478	8,067
Pfam_Annotation	14,591	5,166	8,973
Swissprot_Annotation	14,154	4,767	8,879
nr_Annotation	23,921	10,080	12,036
All_Annotated	24,036	10,144	12,075

Supplementary Table 3. qPCR Primer

Gene	Forward primer (5’-3’）	Reverse primer (5’-3’）
MSTRG.31509	CCTTCGAGTATGGCTTCAA	TCCTTGTATGTTGGTCTTGT
MSTRG.19722	CTTGGACTCGTAAGGAACA	TGGAAGTGGTTATGGATCTG
MSTRG.19723	CCTTCGAGTATGGCTTCAA	TTCAGTCCTTGTATGTTGGT
MSTRG.35242	GCTTGATGGCTGTGTCTT	GCTGCTGATTCTACTTCTGA
evm_000257	CCTACGAATATCTTGCTGTTC	GCTGCTTGGTGCTATCAT
evm_013666	CGGAACTATCAGATGGAACT	ATGTATCGGAGAAGGATTGG
evm_014368	TTCGTCGGACAGGATACC	CTGAGGCAATGGACAAGAA
evm_010324	TCAGCCAAGGAGGTAAGTA	CCACCAGATCCATAATCACT
evm_010325	GCGTGGTAAGAAGAGGAAT	CAGGTGTCGTCAGGAATAC
evm_004429	CTTCCTTGACGACATTCTTG	TACGACAACCTACACAACTT
evm_009930	TCGGTGACAGAAGGAAGT	GTTGCTGGCTAAGATAATGC
evm_013436	GACCGTAGTTCGTGTTGT	ATGACCATCGCTAAGGAAG
EF1	AAAATGGACTCGACTGAACCCC	TCTCCGTGCCAACCAGAAATA
